# The Time for Chronotherapy in Radiation Oncology

**DOI:** 10.3389/fonc.2021.687672

**Published:** 2021-05-11

**Authors:** Luis Bermúdez-Guzmán, Alejandro Blanco-Saborío, Juliana Ramírez-Zamora, Eduardo Lovo

**Affiliations:** ^1^ Robotic Radiosurgery Center, International Cancer Center, San José, Costa Rica; ^2^ International Cancer Center, Diagnostic Hospital, San Salvador, El Salvador

**Keywords:** chronobiology, circadian cycle, radiotherapy, radiobiology, cell cycle, DNA repair

## Abstract

Five decades ago, Franz Halberg conceived the idea of ​​a circadian-based therapy for cancer, given the differential tolerance to treatment derived from the intrinsic host rhythms. Nowadays, different experimental models have demonstrated that both the toxicity and efficacy of several anticancer drugs vary by more than 50% as a function of dosing time. Accordingly, it has been shown that chemotherapeutic regimens optimally timed with the circadian cycle have jointly improved patient outcomes both at the preclinical and clinical levels. Along with chemotherapy, radiation therapy is widely used for cancer treatment, but its effectiveness relies mainly on its ability to damage DNA. Notably, the DNA damage response including DNA repair, DNA damage checkpoints, and apoptosis is gated by the circadian clock. Thus, the therapeutic potential of circadian-based radiotherapy against cancer is mainly dependent upon the control that the molecular clock exerts on DNA repair enzymes across the cell cycle. Unfortunately, the time of treatment administration is not usually considered in clinical practice as it varies along the daytime working hours. Currently, only a few studies have evaluated whether the timing of radiotherapy affects the treatment outcome. Several of these studies show that it is possible to reduce the toxicity of the treatment if it is applied at a specific time range, although with some inconsistencies. In this Perspective, we review the main advances in the field of chronoradiotherapy, the possible causes of the inconsistencies observed in the studies so far and provide some recommendations for future trials.

## Introduction

The Nobel Prize in Physiology or Medicine 2017 was awarded jointly to Jeffrey C. Hall, Michael Rosbash and Michael W. Young for their work on the molecular mechanisms controlling the circadian rhythms (1, 2). The term circadian is derived from the Latin *circa diem* which means “around a day” and was coined by the pioneer physician Franz Halberg (3). Thus, circadian rhythms are daily cycles that control physiological processes at the transcriptional level through networks of genes that oscillate in this 24-hour fashion (4). The circadian transcriptional machinery consists of two transcription factors, CLOCK and BMAL1 (the activators) that heterodimerize and bind to the E-Box sequences of the promoters of ~10-15% of our genes to direct their rhythmic transcription (5, 6). This transcriptional activity peaks during the day but is inhibited at night by the proteins period (PER) and cryptochrome (CRY) (the repressors) (5). Additionally, several kinases and phosphatases regulate the phosphorylation of both activators and repressors, controlling the localization and stability of these integral clock proteins (6).

The central circadian clock, the circadian pacemaker, is found in the hypothalamic suprachiasmatic nucleus (SCN) (7) which exerts control over several aspects of human physiology, including metabolism and sleep regulation. In addition, the SCN is also responsible for storing seasonal day-length information (8), allowing our circadian clock to adapt to seasonal changes in the natural light-dark cycle (9). Mechanistically, the SCN receives information about the time of day through light detected by ganglion cells of the retina and transmitted through the retinohypothalamic tract (RHT). Consequently, the daily patterns of physiology and behavior can be severely altered in blind people due to the lack of photic entrainment. In fact, more than 50% of blind people who lack a conscious perception of light cannot synchronize to the 24-hours day (10).

**Figure 1:**
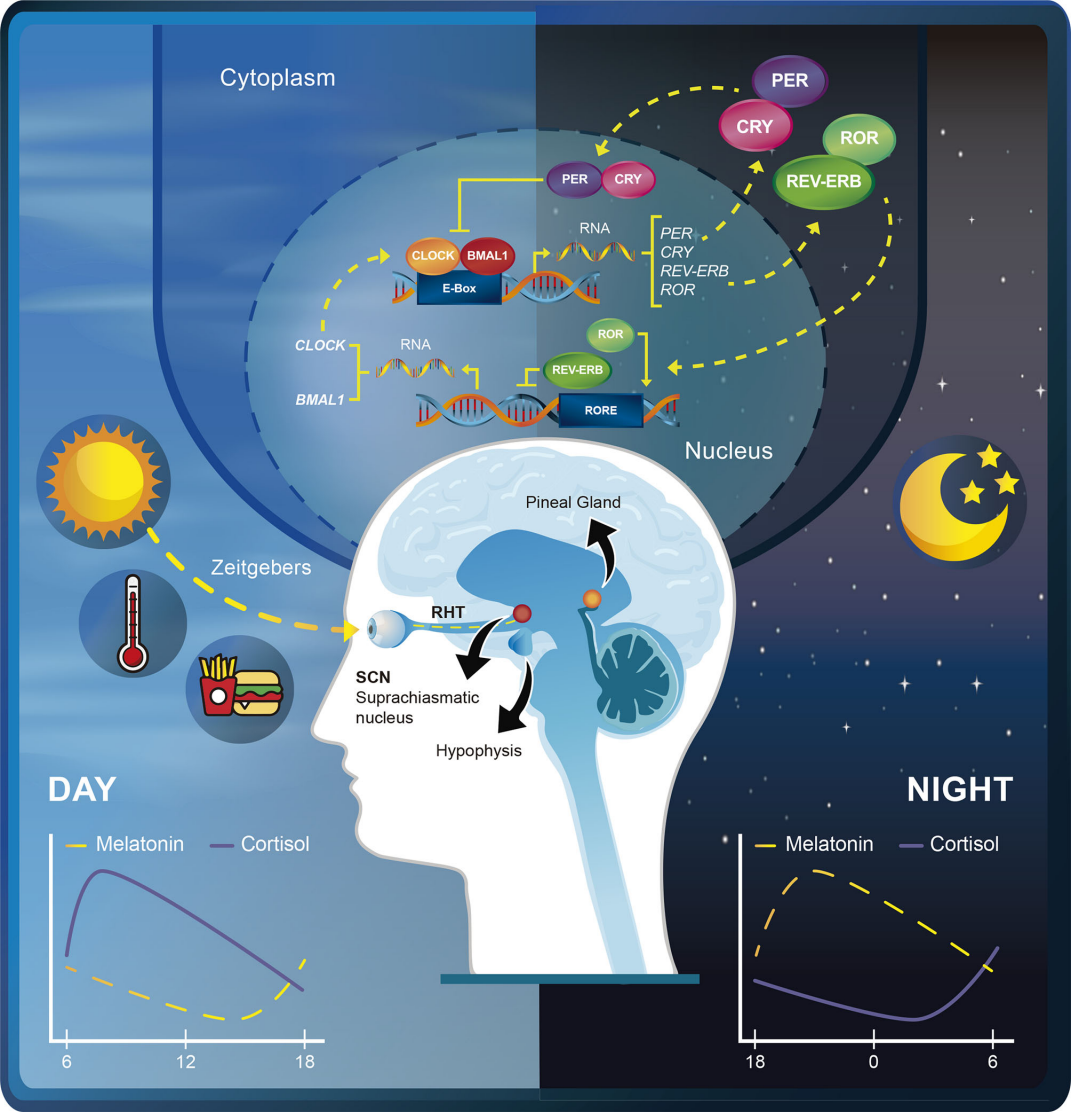
Schematic depiction of the rhythmic expression of the circadian transcriptional machinery. The transcriptional activity of CLOCK-BMAL1 peaks during the day but is inhibited at night by the transcription repressors PER-CRY. RORs activate the transcription of BMAL1 and CLOCK, whereas REV-ERBs repress BMAL1 and CLOCK through retinoic acid-related orphan receptor response element (RORE) binding. Zeitgebers such as light, temperature, and food synchronize the phase of the internal circadian clock relative to the external time. Light is detected by ganglion cells of the retina and transmitted through the retinohypothalamic tract (RHT) to the suprachiasmatic nucleus (SNC). The SCN in turns ensures that the pineal gland rhythmically produces melatonin and drives the release of adrenocorticotropic hormone (ACTH) from the hypophysis. This promotes daily peaks of melatonin and cortisol release at different times of the day.

The phase of the circadian clock (the stage in the cycle relative to external time) is determined by environmental cues named “zeitgebers” (such as light, temperature and food intake). The strength of the stimulus and the circadian phase during which it is applied will determine the response of the circadian clock to zeitgebers (11). Notably, these stimulus can function as “synchronizers” which in turn can reset the body’s circadian clock and place all cells in the same phase of circadian oscillation, in a process called circadian rhythm synchronization (12). Internal representations of the time of day are transmitted to the rest of the body through hormones, the sympathetic/parasympathetic nervous system, and the core body temperature (11). Thus, the central pacemaker can drive peripheral clock rhythms that are under the control of endogenous regulatory factors from the SCN (12). For instance, the SCN ensures that the pineal gland rhythmically produces melatonin (peak levels at night) to promote sleep in diurnal animals. Likewise, the SCN drives the release of adrenocorticotropic hormone (ACTH), which in turn causes an increase in corticosterone release from the adrenal gland in the mornings (13). **Figure 1** summarizes the core components of the circadian clock at the molecular and systemic levels.

## Circadian Cycle in Cancer

### Circadian Cycle and Cancer Susceptibility

More than a decade ago, the International Agency for Research on Cancer (IARC) listed shift work (day/night) as a probable human carcinogen (classified in Group 2A) (14). Nowadays, it is well known that the disruption of the circadian cycle is associated with a higher incidence of different types of cancer (15). Although the association has been established in several epidemiological studies, the causes and factors related to this interruption remain unclear. However, a recent study reported that circadian dysregulation of DNA repair may increase DNA damage and predispose to elevated cancer risk in night shift workers (16). Even when some studies provide compelling information on association between the circadian cycle and cancer, it is not yet clear whether there is any specificity for different types of cancer (17, 18).

Some studies have already demonstrated that polymorphisms in the circadian clock genes are associated with higher cancer risk. For instance, one study demonstrated that several SNPs in different core circadian genes were associated with susceptibility to prostate cancer (19). Similarly, *NPAS2* has been associated with an increased predisposition to sarcoma and breast cancer risk (20, 21). Another study showed a relationship between an increased breast cancer risk and polymorphisms in *CRY2*, *PER2* and *PER1* (22). Polymorphism in *NPAS2*, *PER1* and *PER2* have also been associated to gastric cancer predisposition (23). Three *CRY2* SNPs were also found to be significantly associated with risk of non–Hodgkin’s lymphoma (24). A polymorphism in the *CLOCK1* gene was shown to increase the risk for colorectal cancer development (25). Besides these associations related to cancer susceptibility, the role of the circadian clock in cancer can be analyzed from three different perspectives: 1) the circadian clock disruption as a carcinogenic agent, 2) the circadian control of carcinogenesis and 3) the circadian rhythm as a guide to administer anticancer treatment (26). We will focus on this last aspect for the purpose of this work.

### Circadian Cycle in Cancer Treatment

Five decades ago, Franz Halberg conceived the idea of a circadian-based therapy (chronotherapy) for cancer, given the differential tolerance to treatment derived from the intrinsic host rhythms (27). Since that moment, the idea behind chronobiology has been relevant to understand how time-related events shape our daily biological responses including response to anticancer treatment (28). Different experimental models have demonstrated that both the toxicity and efficacy of over 30 anticancer agents vary by more than 50% as a function of dosing time (29). The rationale for this relies on the fact that time-dependent efficacy of treatments may vary according to three general aspects: 1) the mechanism of action, 2) the pharmacokinetics/metabolism and 3) the variable toxicity depending on circadian rhythms (30). Accordingly, it has been shown that regimens optimally timed with the circadian cycle have jointly improved patient outcomes in terms of tolerance and efficacy of chemotherapeutic treatments, both at the preclinical and clinical level (31, 32). One of the best examples of this is the use of chronochemotherapy for gynecological and genitourinary cancers (33).

## Chronotherapy in Radiation Oncology

### Preclinical Studies

Human cells undergo daily cycles in gene expression, protein levels and enzymatic activity. Accordingly, circadian rhythm-dependent cell cycle progression can produce variations in the response to radiological treatment as cells are most radiosensitive in the G2-M phase (34). Radiation Therapy (RT) is widely used for cancer treatment, but its effectiveness relies mainly on its ability to damage DNA. In fact, radioresistance partly emerges because of efficient and redundant DNA repair capacities (35). It is also known that a two-way connection between DNA repair and cell cycle ensures genomic integrity within cells (36). Notably, the DNA damage response (DRR) including DNA repair, DNA damage checkpoints, and apoptosis is also gated by the circadian clock (37). Thus, the therapeutic potential of circadian-based radiotherapy against cancer is mainly dependent upon the control that the molecular clock exerts on DNA repair enzymes across the cell cycle. In mammalian cells, the ATM/Chk2 signaling pathway is activated by double-strand breaks that are mainly induced by ionizing radiation (IR) (38). The circadian protein PER1 participates in this ATM-Chk2 signaling pathway in response to IR by directly interacting with both proteins (37, 39). Consequently, PER1 is upregulated by radiation and is required for radiation-induced apoptosis (39). **Table 1** summarizes some the most important preclinical studies addressing the effect of the circadian clock on DDR and the toxicity and response to radiation therapy. A better understanding of the control that these clock proteins exert on DRR at the molecular level will provide further insights at the clinical level to develop accurate circadian-based radiotherapy regimes.

**Table 1:** Preclinical studies on the role of the circadian clock in DNA repair and radiotherapy.

Type of study	Model	Hypothesis	Main findings	Reference
*In vitro*	Mouse splenocytes	Day and night variations influence IR-induced DNA damage repair	IR-induced DNA damage is more efficiently repaired during the light phase due to day-time-dependent expression levels of clock-associated genes (especially DNA repair genes).	(40)
*In vitro* *In vivo*	Hair follicles/Transgenic mice	The circadian clock influences the mitotic activity and regeneration of anagen hair follicles.	Hairs grow faster in the morning than in the evening leading to a remarkable time-of-day–dependent sensitivity of growing hair follicles to genotoxic stress. Same doses of γ-radiation caused dramatic hair loss in WT mice when administered in the morning, compared with the evening, when hair loss is minimal.	(41)
*In vivo*	Xenografted BALB/c (nu/nu) mice	Topotecan (TPT) and RT can be chronomodulated to get better results in a model of human nasopharyngeal carcinoma.	The TPT-RT combination was more effective than TPT or RT as single agents. The TPT-RT combination at 15 hours after light onset (HALO) was best and TPT-RT at 3 HALO was worst.	(42)
*In vivo*	mPer2-/- mice	The mPer2 gene functions in tumor suppression by regulating DNA damage-responsive pathways.	mPer2-/- mice show a neoplastic growth phenotype and an increased sensitivity to γ-radiation, manifested by premature hair graying, increased tumor occurrence, and reduced apoptotic response in thymocytes.	(43)
*In vitro*	Human fibroblasts	The cellular response to DNA damage is related to the endogenous expression levels of *PER2.*	Clonogenic cell survival, double-strand break repair kinetics, and TP53 activation were affected in irradiated cells with low endogenous PER2 protein levels (compared to high levels).	(44)
*In vivo*	WT and Per1/2 KO Mice	The circadian system plays regulatory roles in minimizing the IR‐induced cardiotoxicity.	Compared to control mice (day shift), circadian clock disruption either environmentally (rotating shift) or genetically (Per 1/2 mutant) significantly exacerbated post-IR cardiotoxicity.	(45)
*In vivo*	Sprague–Dawley male rats	Per1 and Per2 can increase the radiosensitivity of glioma.	High expression of Per1/2 was associated with increased sensitivity to x-irradiation only in glioma tissue. The high expression of Per1/2 can induce cell cycle arrest and increase tumor sensitivity to x-rays through a p53-dependent mechanism.	(46)

### Clinical Studies

In clinical practice, the time of treatment administration is not usually considered and varies along the daytime working hours. Currently, only a few studies have evaluated whether the timing of radiotherapy (chronoradiotherapy) affects the treatment outcome. Some of these studies have determined that it is possible to reduce the toxicity of radiotherapy if it is administered at a specific time, although this is dependent on the type of cancer (47). Despite this, there are inconsistencies in the literature regarding the treatment outcomes of this approach. However, we consider that part of the inconsistencies derives from important differences in methodology. Additionally, it is well known that the circadian time-dependent interaction between host, cancer and treatment outcome is further impacted by inter-individual differences and clock genes polymorphisms (29).

To date, three studies (47–49) have compiled the conclusions of the main prospective and retrospective studies that have evaluated the effect of time of day with respect to treatment outcome after radiotherapy. From these studies, only three are randomized prospective trials. Most of these studies used different sources of irradiation, used different time intervals for morning/afternoon groups, used symptoms as the primary endpoint, and only few used consensus guidelines to evaluate treatment outcome. This makes it difficult to present definitive claims about the effect of chronoradiotherapy. Therefore, we will highlight the most remarkable aspects of these studies that can be useful for future trials.

The three prospective randomized studies (n = 611) analyzed the effect of time of day on the prevalence of mucositis after radiotherapy delivered in the morning and afternoon. Two of the studies looked at the severity and prevalence of radiation-induced oral mucositis in head and neck cancer but found no clear difference between the two groups (50, 51). However, a consistent trend between both studies was that patients treated in the afternoon exhibited a more rapid progression in the grade of mucositis and the median time to development of grade III/IV mucositis was significantly longer in morning patients (50, 51). What is remarkable from one of these trials (51) is that the study was based on the previous demonstration of a circadian rhythm in the human oral mucosa cell cycle, with most cells in the G1 phase in the morning and M phase at night. Interestingly, a recent retrospective study (n = 617) evaluating the impact of delivery daytime and seasonality of radiotherapy for head and neck cancer found higher acute toxicity for radiotherapy delivered in dark seasonality (each year was divided into dark and light by the March and September equinoxes) (52).

The third prospective randomized study evaluated the prevalence of acute gastrointestinal mucositis in cervical cancer. Interestingly, contrary to what was observed in head and neck cancer, patients in the morning group exhibited a higher prevalence of grade III/IV mucositis than patients treated in the afternoon (53). However, we consider that the endpoint of this retrospective study should be addressed with the current management guidelines for cervical cancer using radiation therapy. Additionally, a different study found that RT in the morning reduces severe hematological toxicity in inoperable cervical cancer patients (using a very similar time range) (54). Taken together, two main aspects can be highlighted from these three prospective randomized studies: 1) all used the Radiation Therapy Oncology Group (RTOG) toxicity criteria and 2) the time range for the morning and afternoon groups was specific and more consistent (morning ranging from 8:00 to 11:00 and afternoon from 15:00 to 20:00).

Four retrospective studies (n = 840) evaluated the effect of chronoradiotherapy on non-small cell lung cancer brain metastases. Two studies found no correlation between time of day and overall survival or local control (55, 56). One study found a trend towards improved median and 2-year overall survival for morning group when a cut-off point of 11:42am was used (57). The last study found that the morning group experienced significantly improved 3-month local control, median overall survival and fewer CNS-related deaths (58). However, the influence of the small sample size on the results of this last study cannot be ruled out. The differences between these retrospective studies may be due to several factors, but we consider that the main drawback in terms of chronotherapy is using a specific time point to separate morning and afternoon groups. We believe that a better option is to define a specific time interval for morning and afternoon cohorts with a significant gap of time between both groups. This would be more appropriate considering that the intention is to translate the biological effects of the circadian cycle at the cellular level on the therapeutic response. Another retrospective study (n = 755) including patients (median age = 66) with multiple brain metastases, found that the time of whole-brain radiotherapy delivery for brain metastases was significantly related to overall survival upon univariate analyzes in females only (59). However, in this study the patients were grouped according to the percentage of sessions (i.e., 100%, 80%, 60%) that they received in one specific time frame. Additionally, patients with many types of primary cancer were included. In fact, when it comes to brain metastases, it has been shown that treatment response, clinical outcomes, and quality of life, are influenced by certain prognostic factors (like number of tumor lesions, functional status, age, comorbidities, etc.) (60, 61). Following this idea and based on these retrospective studies, we recommended to have a cohort of patients as homogeneous as possible when analyzing the impact of chronoradiotherapy on patient outcomes.

For primary brain cancer, few studies have addressed the utility of chronoradiotherapy. One study demonstrated that time-of-day-dependent sensitivity to radiation was different in normal and cancerous cell lines of the central nervous system based on PER2 expression (48). Both rat and human GBM cell lines were more affected by radiation at different times compared to SCN cells, suggesting that timing of radiation could be optimized to improve detrimental effects on healthy tissue, while still providing effective antitumoral doses. A retrospective study (n = 109) evaluating the impact on chronoradiotherapy in high grade gliomas found no difference in overall survival and progression free survival for patients treated in the morning or afternoon (62). However, in terms of timing, patients were included in the morning group if ≥50% of fractions were delivered before 12:00 h.

Two different studies (n = 1275) analyzing toxicity associated with breast cancer radiotherapy found opposite results. The first showed a higher incidence of worse reactions in the morning (63) (in both retrospective and prospective cohorts) while the other reported a higher incidence of grade 2 skin reactions in the afternoon (after 15:00 h) (64). Notably, the clinicopathologic characteristics were relatively well balanced between the treatment groups in the latter study. Additionally, it is also remarkable that the former study showed that an increased late effect in the group receiving morning radiotherapy was associated with carriage of a variable number tandem repeat (4/4 genotype) in *PER3* and a SNP (rs131116075) in the *NOCT* gene (AA genotype) (63).

One retrospective study (n = 409) in patients undergoing definitive high-dose RT for prostate cancer (median 78 Gy) found that evening RT may lead to more gastrointestinal complications, especially in patients older than 70 years old (65). However, the specific time point to define daytime treatment (before 5 PM) is very broad in our opinion. Additionally, it should be considered that for prostate cancer, there must be an adequate patient preparation (rectum and bladder) to reduce the movement of the gland during or between sessions of radiotherapy as that may affect treatment outcome. Another study (n = 168) in patients with localized prostate cancer found that lower urinary tract symptoms were significantly ameliorated in patients who received proton beam therapy in the morning (before 10:30 AM) (66).

A retrospective study (n = 155) in patients with locally advanced rectal cancer found that those who received the majority of their radiotherapy fractions after 12:00 pm were more likely to show a complete or moderate pathological response and improved nodal downstaging (67). Notably, less tumor response was reported in females when compared to males, but this may be caused by gender imbalance (45 females versus 110 males). Additionally, there was no defined time range, but patients were separated according to the percentage of fractions received after a specific time point (12:00 pm).

## Discussion and Future Directions

**Figure 2:**
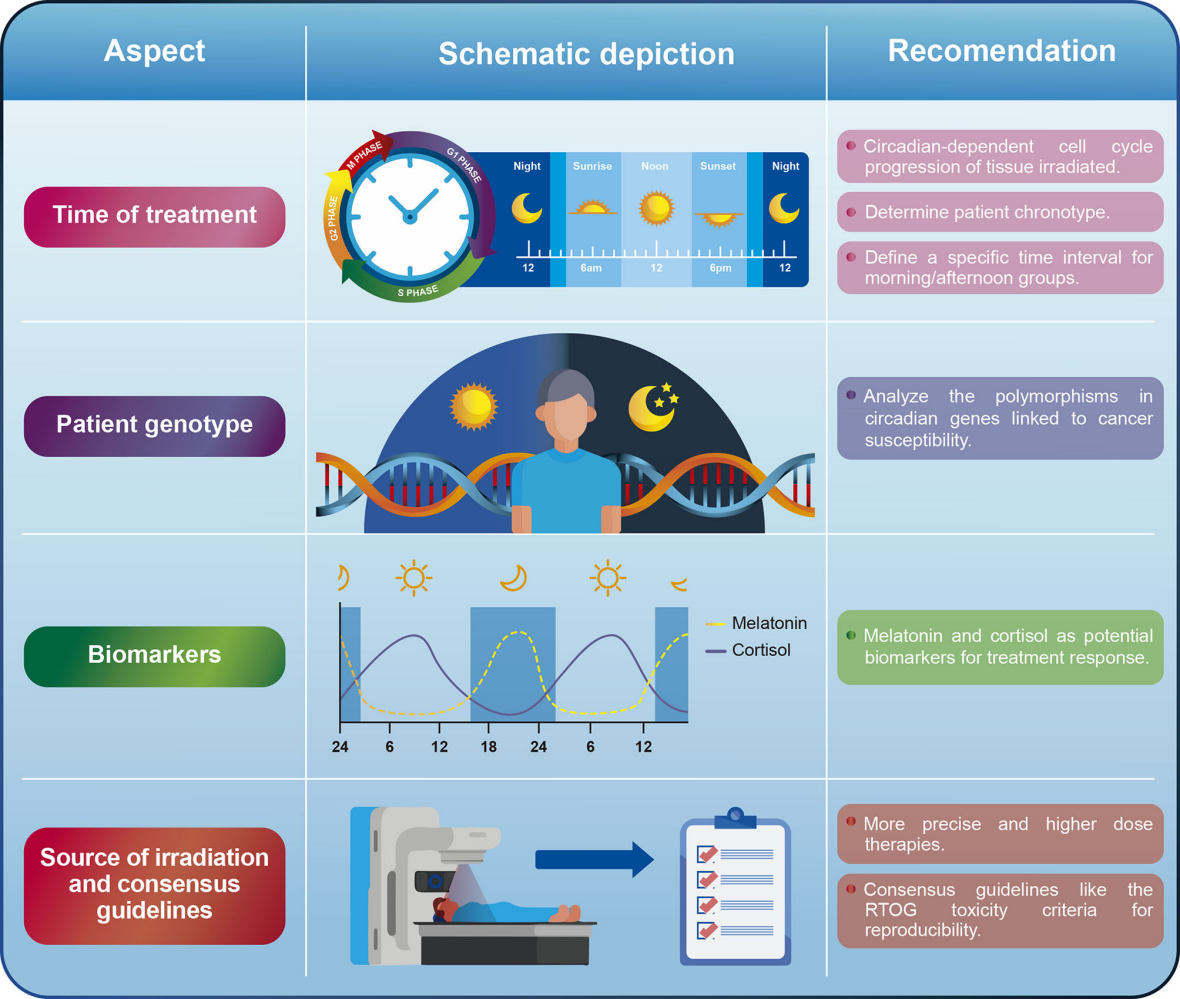
General recommendations for some of the aspects that must be improved or addressed in chronoradiotherapy.

Although the evidence so far indicates that chronoradiotherapy could represent a promising approach in clinical practice, some variables still need to be standardized to extend its use. Additionally, more basic research is needed to guide potential clinical trials in different types of cancer, especially those that rely more on radiological treatment. In the next section, we will discuss some aspects that must be either improved or taken into consideration for future chronoradiotherapy trials (**Figure 2**).

### Time of Treatment

For most studies so far, there is a lack of consensus when defining a time range for morning and afternoon cohorts. Although we recognize that it might be difficult to reach this consensus, we suggest to define time ranges similar to the ones reported in the head and neck cancer retrospective studies (50, 51). Short periods of time for the morning (i.e., 8:00-10:00am) and afternoon (i.e., 16:00-18:00) groups would be useful to evaluate whether it is possible to capture significant biological differences derived from the circadian rhythm. Likewise, separating both groups by at least 4-6 h would allow the differences observed between groups to be attributed to the circadian cycle with greater certainty. We do not recommend using a specific time point to separate patients in the morning/afternoon arms. It can also be useful to analyze the existing evidence on the circadian rhythm dependent cell cycle progression of the tissue that is being irradiated to guide clinical trials. This approach was shown to be useful for head and neck cancer, where a circadian rhythm in the human oral mucosa cell cycle was demonstrated, leading to the hypothesis that morning radiotherapy would cause less oral mucositis (51).

A complementary approach could be to define the chronotype of the patients under study in prospective trials. The chronotype is a representation of the patient’s circadian rhythm and refers to preferences for timing of sleep and wakefulness. Early-type subjects (commonly known as larks) naturally wake up and fall asleep earlier than late-type individuals (known as night owls) (68). Identifying patient’s chronotypes can be done *via* different ways including survey, either by using the well-known Morningness-Eveningness Questionnaire (MEQ) (69) or the Munich ChronoType Questionnaire (MCTQ) (70). Determining the chronotype is important since the internal time is not the same between individuals as their endogenous circadian clocks have different phase relationships with respect to external clock time (71). None of the studies carried out so far evaluating the effect of chronoradiotherapy has determined the chronotype of the patients. In contrast, some studies have found a relationship between the patients’ chronotype and chemotherapy-associated toxicity. For example, one study found that late chronotypes are associated with neoadjuvant chemotherapy-induced nausea and vomiting in women with breast cancer (72). For this reason, we encourage to carry out prospective trials that consider patients’ chronotypes.

### Patient Genotype

Different clock genes polymorphisms have been associated with cancer susceptibility, especially colorectal (25), breast (22), and gastric cancer (23). Although the involvement of these genes in different cellular pathways is known, little is known about their influence on the response to cancer treatment, especially in radiotherapy. Circadian clock PER proteins (PER1, PER2, and PER3) are important repressors of the transcriptional activity of the CLOCK/BMAL1 complex. Additionally, PER1 participates in the ATM-Chk2 signaling pathway in response to IR by directly interacting with both proteins (37, 39) as it is upregulated by radiation and required for radiation-induced apoptosis (39). So far, only one study evaluating chronoradiotherapy in breast cancer has shown that certain alleles of two circadian rhythm genes (*PER3* and *NOCT*) predict worse outcome in the morning group (63). Although it may be somewhat difficult to incorporate into clinical practice, future trials should evaluate the polymorphisms of those clock genes that have been linked to cancer susceptibility and treatment response. Thus, when considering the genotype of patients, it could be determined which polymorphism are associated with a better or worse response to chronoradiotherapy.

### Biomarkers

The variations in hormonal levels during the day are also closely linked to the circadian cycle. For example, serum cortisol shows low values at night, a peak early in the morning (7:00-8:00am) and decreasing values during the day (73). On the other hand, serum melatonin presents high values at night and extremely low values during the day (74). It has been shown that stress hormones can increase DNA damage and alter transcriptional regulation of the cell cycle (75). For instance, long exposures (24 h) in dose-response experiments with norepinephrine or epinephrine induced significant increases in DNA damage in treated cells compared to that of untreated cells (76). Likewise, acute exposure to cortisol and norepinephrine significantly increased levels of ROS/RNS and DNA damage in breast cancer cell lines (77). One study in patients with metastatic breast cancer (n = 104) found that the variability in the diurnal cortisol rhythm is a significant predictor of survival time (78).

On the other hand, it has been demonstrated that melatonin exerts some anticancer activity especially mediated by interfering with various cancer hallmarks (79). It is well known that melatonin modulates DNA damage response and DNA repair pathways (80). For instance, melatonin induces phosphorylation of p53 inhibiting cell proliferation, preventing DNA damage accumulation of both normal and transformed cells (81). Additionally, melatonin showed to enhance the effects of radiotherapy (82), by sensitizing cancer cells to ionizing radiation (83, 84). Likewise, pre-treatment with melatonin was also shown to ameliorate harmful effects of irradiation-induced oxidative damage in rat peripheral blood (85). Notably, a meta-analysis of human trials using melatonin as adjunct treatment concurrent with chemotherapy or radiotherapy found that melatonin significantly improved tumor remission, 1-year survival, and alleviation of radiochemotherapy-related side effects across different types of cancer (86). Another study (n = 30) showed that survival at 1 year was significantly higher in patients treated with RT plus melatonin than in those receiving RT alone (87). Additionally, RT or steroid therapy-related toxicities were lower in patients concomitantly treated with melatonin. Taken together, these hormones could be used as response or prognostic markers for chronoradiotherapy since they have been shown to play an important role in DNA repair and response to radiation therapy. However, none of the studies carried out so far evaluating the effect of chronoradiotherapy have also measured the serological levels of these hormones to assess whether they can serve as biomarkers correlating to treatment response.

### Clock-Modulating Compounds

Many studies have identified several small-molecule agonists and antagonists for clock-related proteins, especially for CRY, ROR and REV-ERB (88, 89). For instance, a recent study showed that that two agonists of REV-ERBs (SR9009 & SR9011) are lethal to cancer cells and oncogene-induced senescent cells but have no effect on the viability of normal cells (90). Another study identified a small molecule called KL001 that specifically interacts with CRY (91). This molecule prevented proteasomal degradation of CRY, resulting in lengthening of the circadian period. Other studies have employed high-throughput chemical screening to identify novel clock-modulating compounds. For example, from a screening of over 1,000 small molecules using an FDA-approved drug library and the International Drug Collection, 5% of the drugs screened altered the circadian period (92). Other studies using high‐throughput screening and circadian luciferase reporter assays have found several compounds targeting the circadian clock out of thousands of molecules (93–95). Future preclinical trials evaluating the effect of chronoradiotherapy in different types of cancer should consider the use of circadian-modulating compounds to prove their potential as adjuvant therapy. Perhaps in the future, clinical trials will be able to optimize the effects of circadian-based radiotherapy with the use of these modulating compounds.

### Source of Irradiation and Consensus Guidelines

Although it could be considered that different irradiation sources might not have a significant impact on chronoradiotherapy, we suggest that new trials should evaluate the use of more precise and higher-dose therapies as optimal doses can be directed to the tumor without causing greater toxicity to adjacent organs. In fact, most studies to date have evaluated the effect of chronoradiotherapy on the toxicity generated by the treatment in the surrounding healthy tissue but few studies have found a direct effect on local tumor control. We believe that the use of Stereotactic Radiosurgery (SRS) (a form of radiation therapy that focuses a high dose of energy on a small area of ​​the body) could be an interesting approach to evaluate chronoradiotherapy. For instance, it has been shown that SRS using Cyberknife generates better local tumor control in some types of cancer (96–99). We consider that the high doses and high precision provided by SRS could be a promising approach to assess tumor response to chronoradiotherapy given the fact that the main target of radiation will be the tumor tissue and not the surrounding tissue. In addition, using SRS ensures shorter treatment schedules (1-2 weeks versus 4-8 weeks or more), reducing the possible effects that other variables apart from timing have on the treatment outcome.

Another important aspect that must be considered is the consensus guidelines used to assess the toxicity following radiation therapy. Only few studies evaluating the effect of chronoradiotherapy have used consensus guidelines such as the toxicity criteria of the Radiation Therapy Oncology Group (RTOG) (100) and The Response Evaluation Criteria in Solid Tumors (RECIST) (101). Future trials should seek to use these or other recognized guidelines in order to make the results obtained between studies more comparable.

## Conclusion

Although we are far from having some general guidelines, chronoradiotherapy represents an approach that deserves to be studied further given the cumulative evidence on the reduced toxicity of circadian-based radiotherapy. However, although the trials carried out so far have shown that it is possible to reduce the toxicity associated with radiotherapy in a time-of-day-dependent manner, many inconsistencies persist due to the lack of guidelines that standardize this practice. We hope that this perspective will provide some new insights and recommendations that guide future clinical trials evaluating the impact of chronoradiotherapy not only in terms of toxicity but also tumor control in different types of cancer.

## Data Availability Statement

The original contributions presented in the study are included in the article/supplementary material. Further inquiries can be directed to the corresponding author.

## Author Contributions

LB-G. conceived the idea and wrote the manuscript with input from all authors. AB-S revised the manuscript. JR-Z revised the manuscript. EL revised the manuscript and managed the project. All authors contributed to the article and approved the submitted version.

## Funding

This work was funded by the International Cancer Center, San José, Costa Rica/San Salvador, El Salvador.

## Conflict of Interest

The authors declare that the research was conducted in the absence of any commercial or financial relationships that could be construed as a potential conflict of interest.
